# Author Correction: Transcriptional read-through of the long non-coding RNA SVALKA governs plant cold acclimation

**DOI:** 10.1038/s41467-019-13269-0

**Published:** 2019-11-08

**Authors:** Peter Kindgren, Ryan Ard, Maxim Ivanov, Sebastian Marquardt

**Affiliations:** Department of Plant and Environmental Sciences, Copenhagen Plant Science Centre, University of Copenhagen, Bulowsvej 34, Frederiksberg, 1871 Denmark

**Keywords:** Long non-coding RNAs, Plant sciences, Plant molecular biology

Correction to: *Nature Communications* 10.1038/s41467-018-07010-6, published online 01 November 2018.

The original version of this Article contained an error in the Data availability statement. The accession code for TSS-seq data was incorrectly indicated as ‘GSE113677’ but should have read ‘GSE119304’. This has been corrected in both PDF and HTML versions of the Article.

