# Supplementary material for: Transcriptional read-through of the long non-coding RNA SVALKA governs plant cold acclimation
Source: Nat Commun. 2018 Nov 1;9:4561. doi: 10.1038/s41467-018-07010-6 (PMC6212407; doi:10.1038/s41467-018-07010-6)
Supplement: Supplementary file 1 — Supplementary Information [file 41467_2018_7010_MOESM1_ESM.pdf]

Supplementary Information

Kindgren et al.

Supplementary Figure 1-13

Supplementary Data 1: Regulated TSS in response to cold

Supplementary Data 2: TSS-seq stats

Supplementary Data 3: Genotypes used in this study

Supplementary Data 4: Oligos used in this study

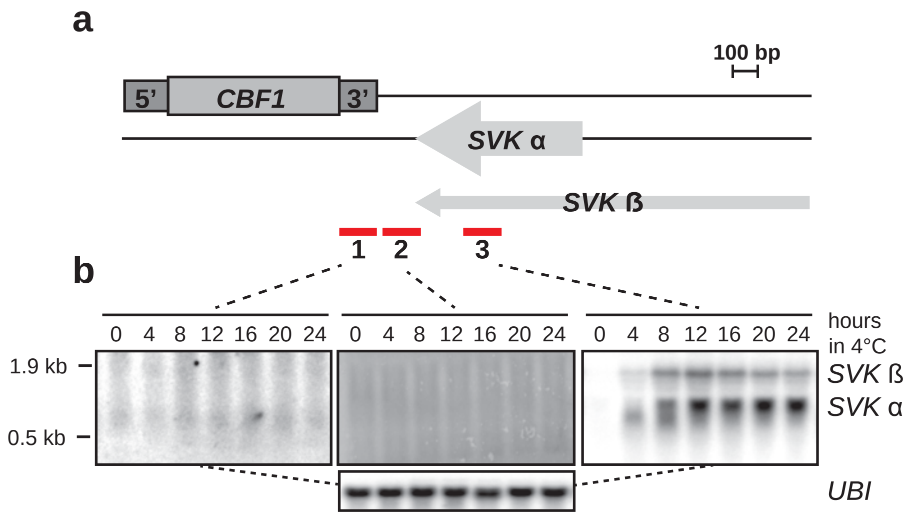

Supplementary Figure 1. Stable *SVK* transcription does not reach the 3'UTR of *CBF1*.
a) Graphical representation of the *CBF1-SVK* genomic region. The probes used in b) are shown with red lines.
b) Representative Northern blots of a cold exposure time series in WT. Blots were repeated with three biological replicates with similar results. Presented are results from the same membrane hybridized with the different probes shown in a). *SVK* transcripts could only be found with probe 3. For probe 1 and 2, membranes were exposed for twice the time as probe 3. No signal (i.e. stable transcripts) was detected further downstream of the identified polyA signal of *SVK*. *UBI* was used as loading control. Uncropped blots can be found in the Source Data file.

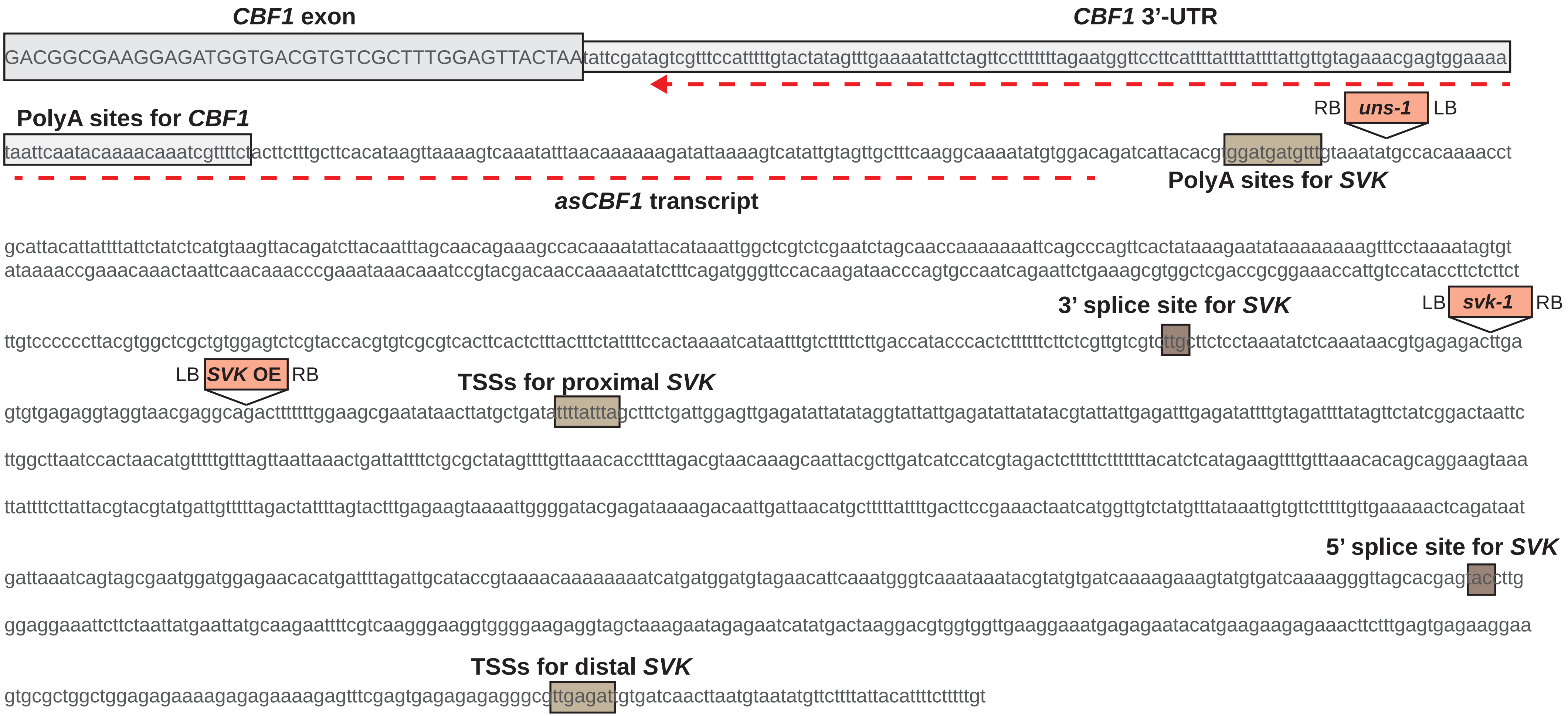

Supplementary Figure 2. Graphical representation of *SVK* RACE analysis.
The sequence shows the sites of *CBF1* PolyA sites and *SVK* TSS and PolyA sites with single-base resolution. The *asCBF1* transcript is indicated with a red dashed arrow. The insertion sites of the T-DNA lines used in this study is indicated.

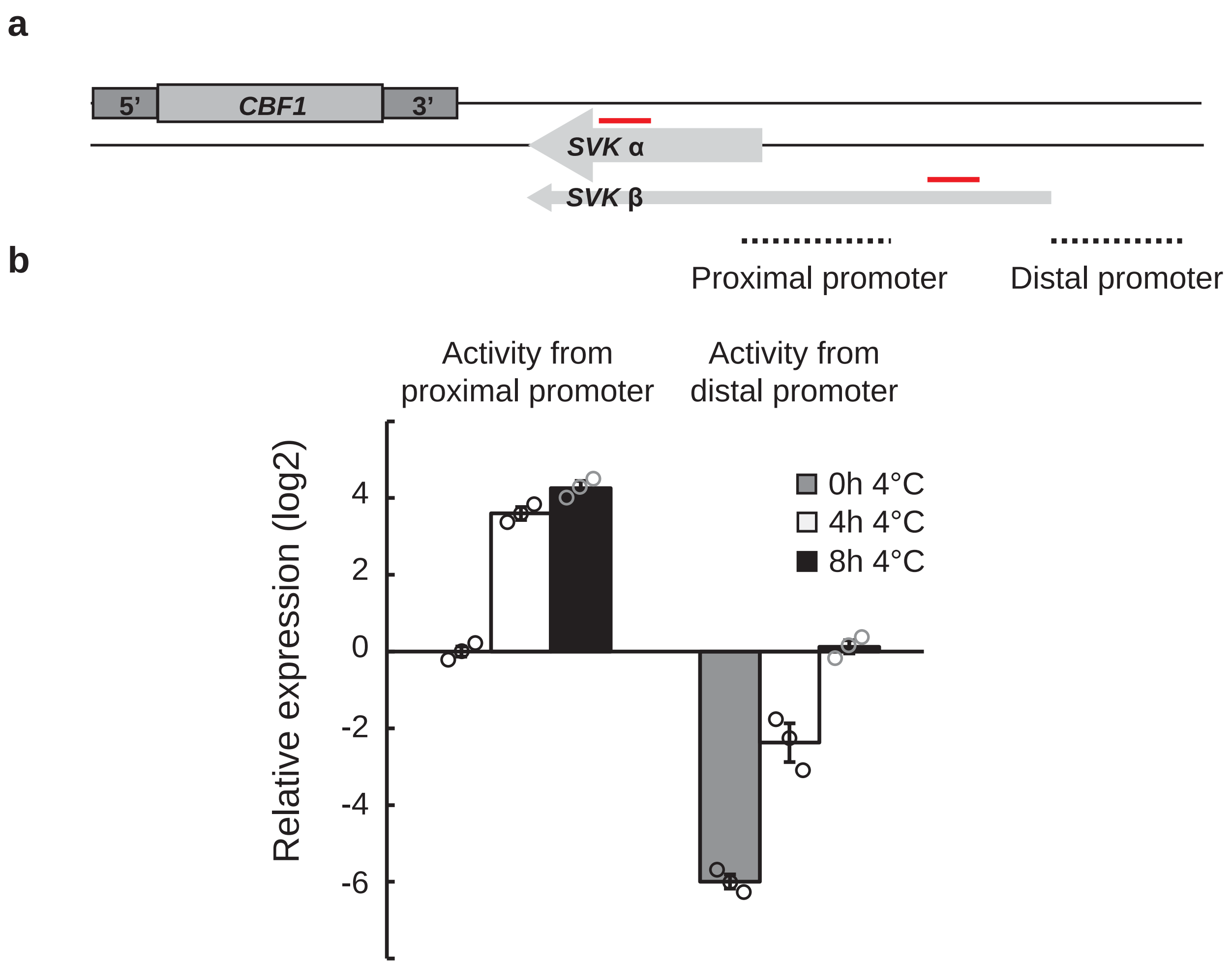

Supplementary Figure 3. Promoter usage of *SVK*.
a) Graphical representation of the *CBF1-SVK* genomic region. The red lines indicate the position of the qPCR probes in b).
b) RT-qPCR with probes detecting transcripts from the proximal and distal promoter of *SVK*. Bars represent mean (grey: 0h 4°C, white: 4h 4°C, black: 8h 4°C, ± SEM) from three biological replicates (rings). The relative level of *SVK* transcripts were normalized to the level at 0h 4°C for the proximal probe. The results are presented with a log 2 scale and show that the level of transcription from the proximal promoter was at a much higher level compared to the activity from the distal promoter. Thus, we named the proximal transcripts *SVK* α, and the transcripts from the distal promoter *SVK* β. Both classes of transcripts showed a response to cold treatment. Source data are provided as a Source Data file.

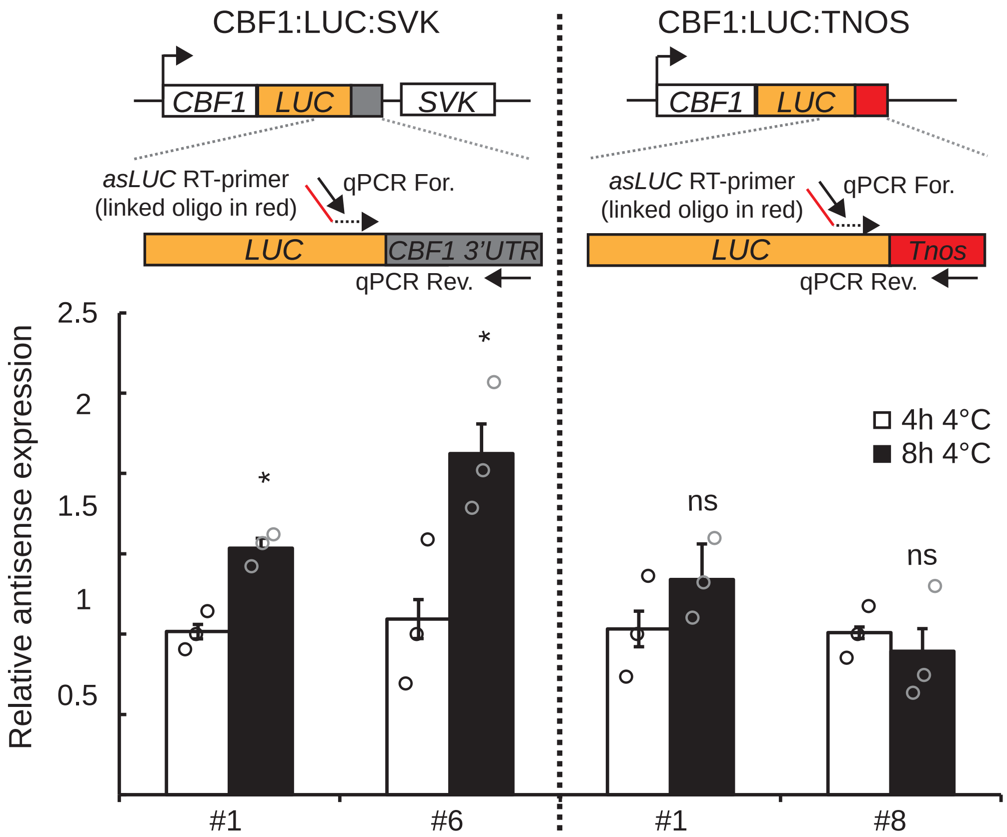

Supplementary Figure 4. RT-qPCR of antisense transcription from *LUC* constructs.
RT-qPCR of antisense transcripts in response to cold exposure in two independent lines from each *LUC* construct. Bars represent mean (white: 4h 4°C, black: 8h 4°C, ± SEM) from three biological replicates (rings). The relative level of transcripts was normalized to the level at 4h 4°C for each probe. Statistical significant differences were determined with Student's t-test (*$p<0.05$). The primers used are indicated in the graphical description of the constructs (above the graph). cDNA was generated with a oligo-linked RT-primer that anneals to the 3'-end of the *LUC* gene to strand-specifically get antisense transcripts of respective lines without genomic DNA contamination.

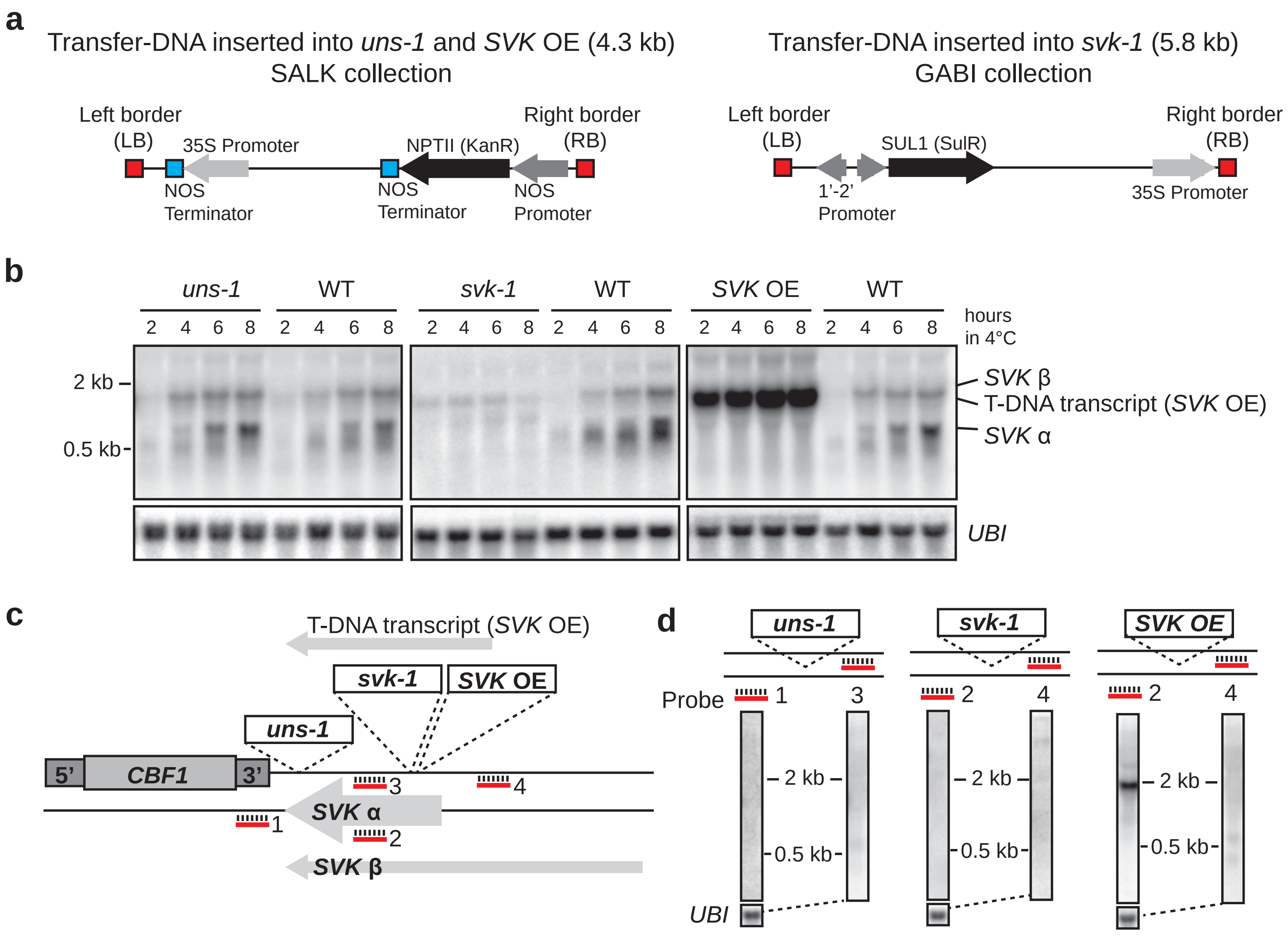

Supplementary Figure 5. Characterization of T-DNA lines that effect *SVK*.
a) Graphical representation of the T-DNA that gets inserted in the mutants used in this study. T-DNA lines from the SALK collection include a 35S promoter close to the left border of the inserted DNA sequence. This promoter drives the expression of the T-DNA transcripts seen in the *SVK OE* mutant. In addition, the Kanamycin resistance in SALK lines is driven by the NOS promoter close to the right border of the T-DNA. For lines form the GABI collection, the SulR resistance gene is driven by the bidirectional 1'-2' promoter. It also includes a 35S promoter close to the right border of the T-DNA.
b) Representative Northern blots of *SVK* expression in T-DNA mutants (using probe 2 in c)) following cold exposure. Blots were repeated with three biological replicates with similar results. *UBI* is used as loading control. Uncropped blots can be found in the Source Data file.
c) Graphical representation of insertion position of T-DNA lines and probes used. Probe 1 and 2 detect *CBF1* antisense transcripts (i.e. *asCBF1* and *SVK*, respectively). Probe 3 and 4 detects sense transcripts downstream of *CBF1*.
d) Representative Northern blots of upstream and downstream probe from the T-DNA insertion site. Blots were repeated with three biological replicates with similar results. For *uns-1* and *svk-1*, no T-DNA originating transcripts could be detected. For *SVK OE*, a strong signal could be detected originating from within the T-DNA sequence and continuing over the *SVK* sequence. However, no signal could be detected for upstream of the insertion site. The lack of signal of *asCBF1* suggests that all stable *SVK OE* transcripts are terminated at the endogenous *SVK* polyA signal.

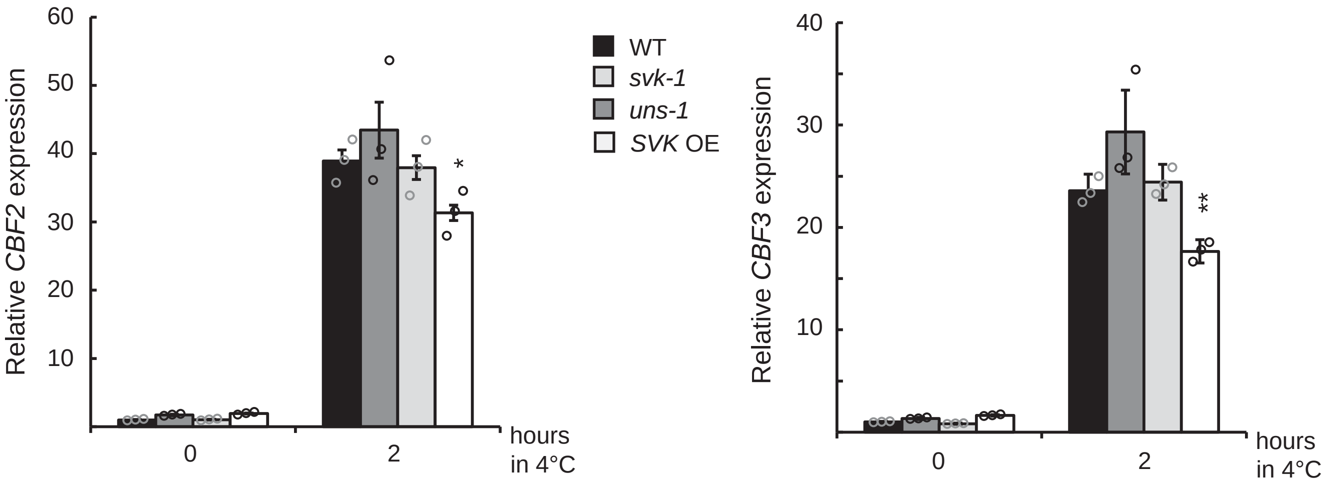

Supplementary Figure 6. *CBF2* and *CBF3* expression in T-DNA lines that effect *SVK*.
Relative *CBF2* (left panel) and *CBF3* (right panel) expression determined by RT-qPCR in WT and mutants that effect *SVK* following exposure to cold. Bars represent mean (black: WT, light grey: *svk-1*, dark grey: *uns-1*, white: *SVK OE*, ± SEM) from three biological replicates. The relative level of *CBF2* and *CBF3* transcripts were normalized to the level in WT in control conditions. Statistical significant differences were determined with Student's t-test (*p<0.05, **p<0.01).

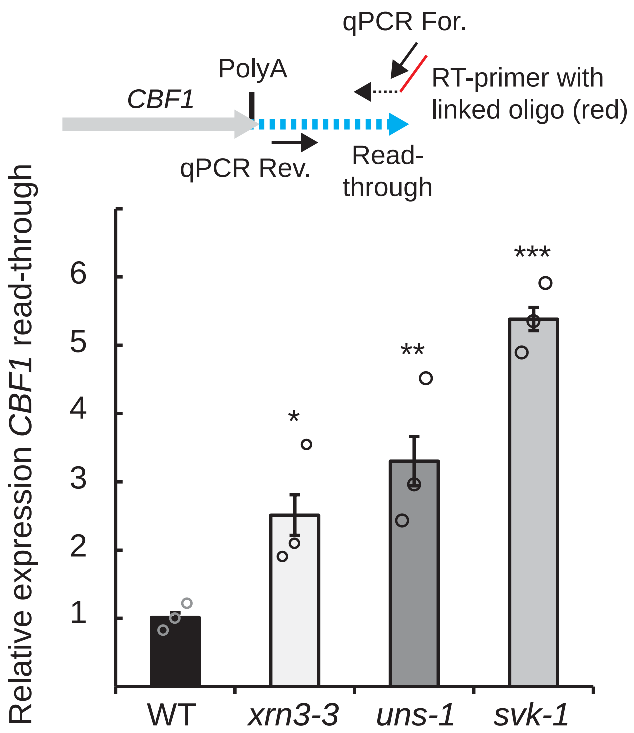

Supplementary Figure 7. Quantification of *CBF1* read-through.
RT-qPCR of *CBF1* read-though after 8 hours of cold exposure in WT, *xrn3-3*, *uns-1* and *svk-1*. We used the *xrn3-3* mutant as a positive control. XRN3 is thought to represent the 5'-to-3' torpedo exonuclease mediating transcriptional termination in Arabidopsis and the *xrn3-3* mutant accumulate 3'-extensions of transcription units. Primers used are shown in the upper panel. cDNA was generated with a oligo-linked RT-primer that annealed downstream of the *CBF1* polyA signal. This ensures strand-specificity. qPCR was run with a forward primer annealing to the linked adapter of the RT-primer and a reverse primer that annealed just downstream of the *CBF1* polyA signal. Bars represent mean (± SEM) from three biological replicates (rings). The relative level of transcripts was normalized to the level in WT. Statistical significant differences were determined with Student's t-test (*p<0.05, **p<0.01, ***p<0.001).

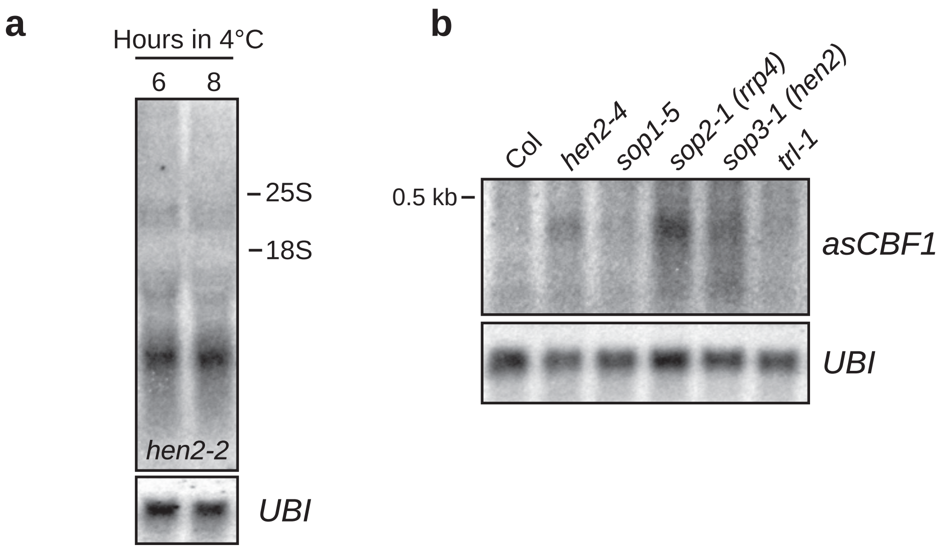

Supplementary Figure 8. Characterization and mutant analysis of *asCBF1*.
a) Representative Northern blot of a cold exposure time series of *hen2-2* showing the complete membrane. Blots were repeated with three biological replicates with similar results. UBI is used as loading control.
b) Representative Northern blot of cold exposure (4h 4°C) of nuclear exosome mutants. Blots were repeated with three biological replicates with similar results. *UBI* is used as loading control. Uncropped blots can be found in the Source Data file.

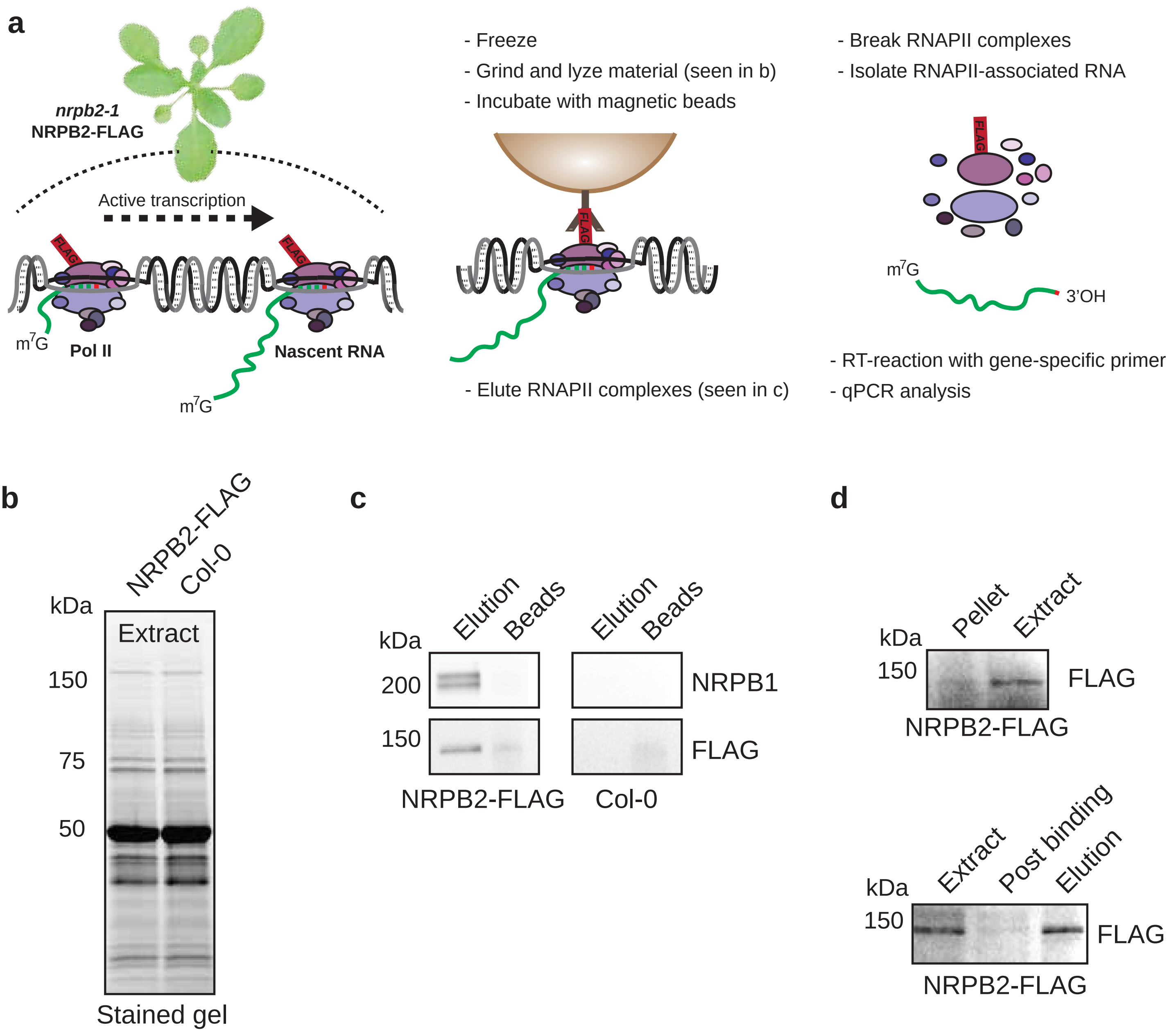

Supplementary Figure 9. Purification of nascent RNA.
a) Flow-chart of purification of nascent RNA from Arabidopsis. More details about the purification procedure can be found in material and methods.
b) Input material for purification of RNAPII complexes visualized from a stain-free TGX-gel. 5 grams of 10-day old seedlings were used for each extraction. Extraction buffer was added to ground powder and used for FLAG-IP.
c) Western blots of elution from magnetic beads with Anti-FLAG and Anti-NRBP1 antibodies. From the NRPB2-FLAG line, both FLAG and NRBP1 were eluted indicating that RNAPII complexes were still assembled in the elution. Most of the RNAPII complexes bound to beads were eluted since no signal was detected from the remaining bead fraction. No signal could be detected in the non-FLAG genotype (Col-0). Uncropped blots can be found in the Source Data file.
d) Western blots to control the purification of RNAPII complexes monitored with Anti-FLAG. Most NRBP2-FLAG signal was found in the extract (upper panel) and eluted from the beads (lower panel).

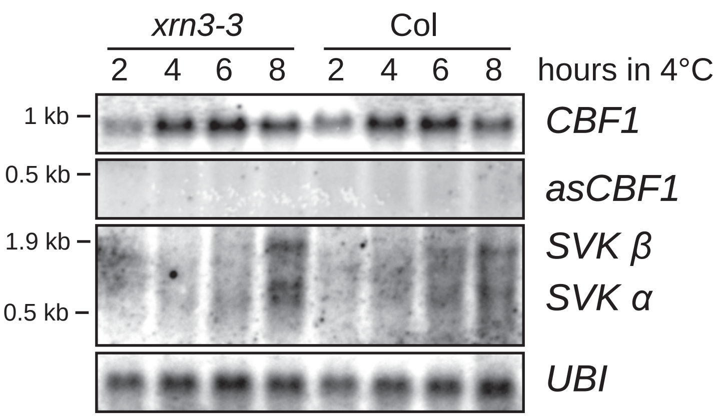

Supplementary Figure 10. Analysis of the *xrn3-3* mutant.
Representative Northern blot of a cold exposure time series of WT and *xrn3-3*. Blots were repeated with three biological replicates with similar results. *CBF1* expression is not affected in the *xrn3-3* mutant and no *asCBF1* signal could be detected. *UBI* is used as loading control. Uncropped blots can be found in the Source Data file.

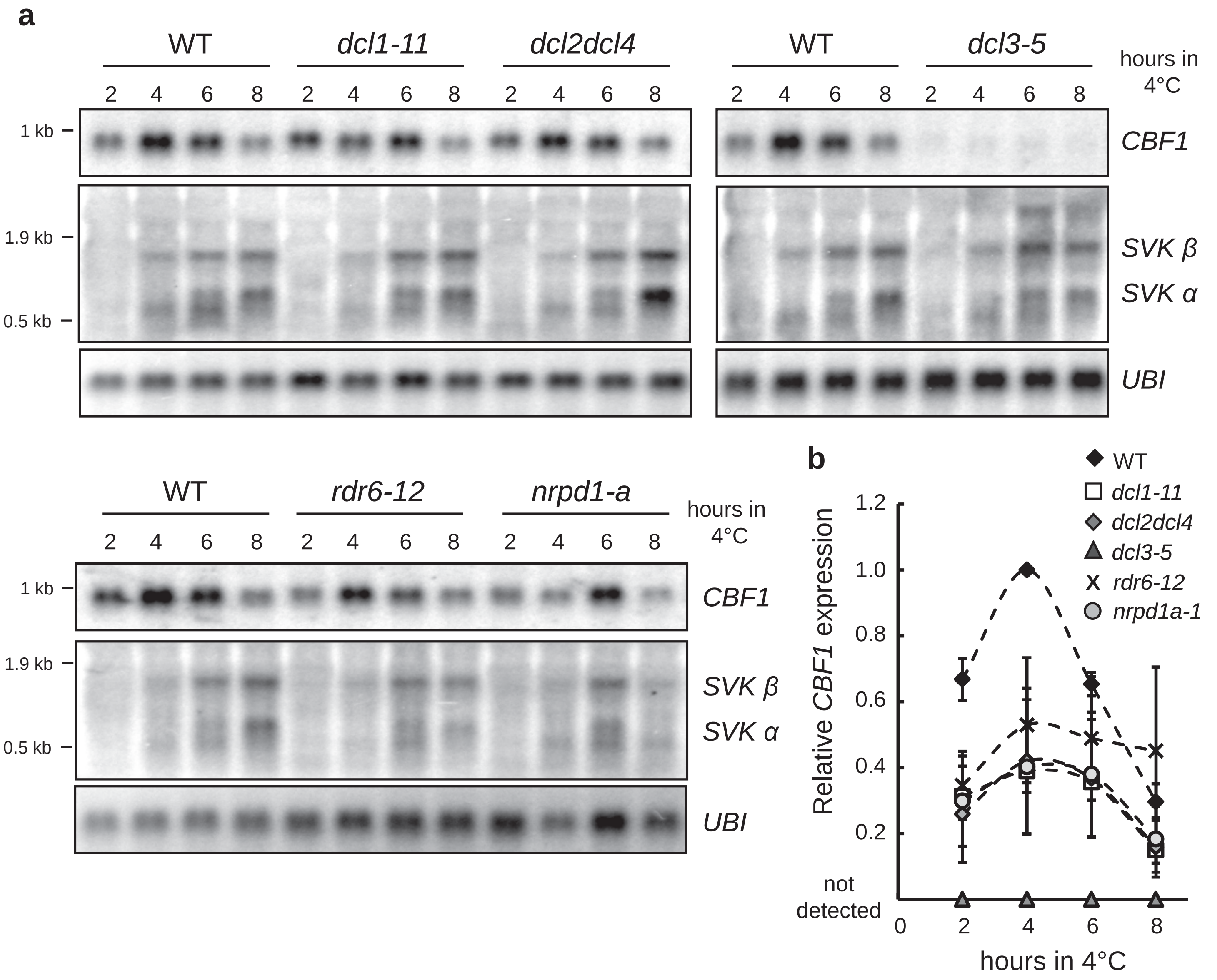

Supplementary Figure 11. siRNA is not involved in *SVK*-mediated repression of *CBF1*.
a) Representative Northern blots of a cold exposure time series of mutants in different siRNA pathways. Blots were repeated with three biological replicates with similar results. *UBI* is used as loading control. Uncropped blots can be found in the Source Data file.
b) Quantification of relative *CBF1* expression after different times of cold exposure in WT and siRNA mutants (black tilted squares: WT, white squares: *dcl1-11*, grey tilted squares: *dcl2dcl4*, dark grey triangles: *dcl3-5*, crosses: *rdr6-12*, light grey circles: *nrpd1a-1*). Northern blot signal intensity from three biological replicates were normalized to their *UBI* signal and further normalized to the relative *CBF1* level in WT after 4h at 4°C.

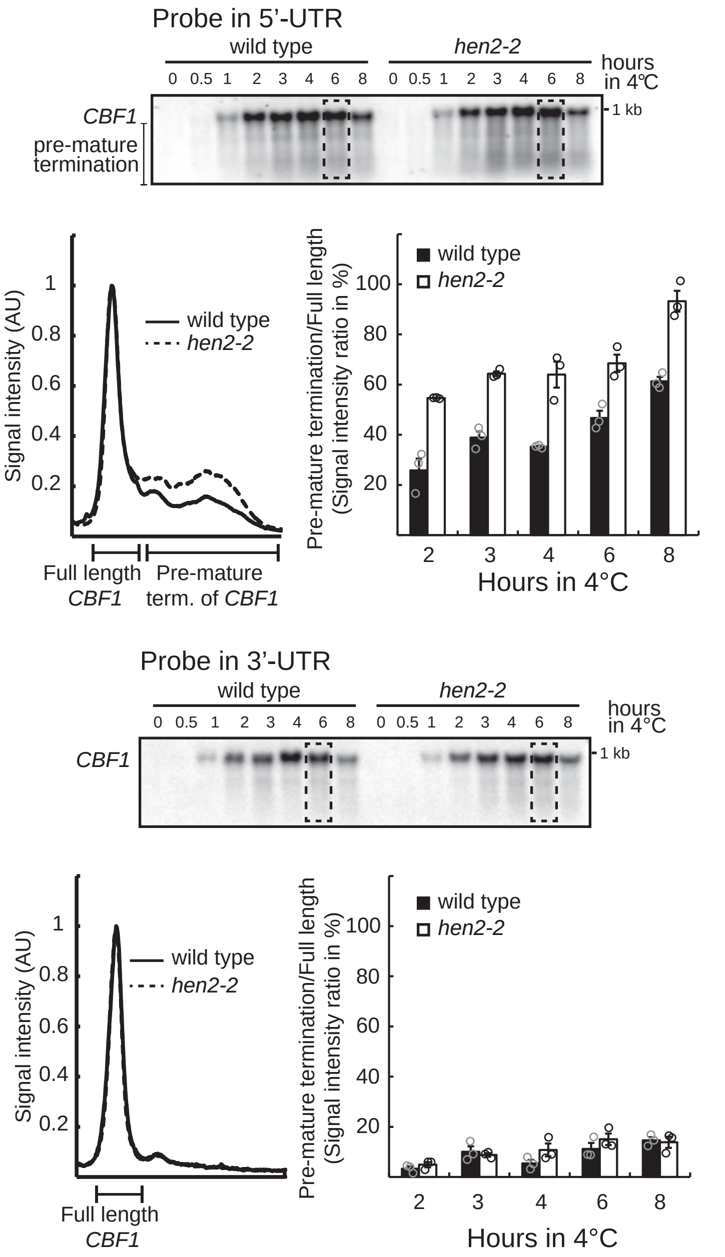

Supplementary Figure 12. Characterization of the mechanism of *SVK*-mediated repression of *CBF1*. Detection of pre-maturely terminated *CBF1* determined by Northern blot in Col-0 (black) and *hen2-2* (white). A probe in the 5'-UTR of *CBF1* detected an increased fraction of pre-terminated *CBF1* (upper panel). A visualization of the signal intensity across the boxed square indicated a higher fraction of pre-terminated *CBF1* in the *hen2-2* mutant. Quantification of all lanes with three biological replicates showed an increase of terminated *CBF1* with increased duration of cold exposure. The combined signal from pre-terminated *CBF1* was represented as a percentage to the signal from full length *CBF1* for each time point. At all time-points the signal from *hen2-2* was higher than Col-0. A probe in the 3'-UTR showed small differences between Col-0 and *hen2-2* (lower panel). Source Data are provided as a Source Data file.
