# Supplementary material for: Transcriptional read-through of the long non-coding RNA SVALKA governs plant cold acclimation
Source: Nat Commun. 2018 Nov 1;9:4561. doi: 10.1038/s41467-018-07010-6 (PMC6212407; doi:10.1038/s41467-018-07010-6)
Supplement: Supplementary file 7 — Description of Additional Supplementary Files [file 41467_2018_7010_MOESM7_ESM.docx]

**Title:** Supplementary Data 1.
**Description:** Significantly up- and down-regulated TSS peaks in response to 3 of hours of 4°C.

**Title:** Supplementary Data 2.
**Description:** Statistics for TSS-seq libraries.

**Title:** Supplementary Data 3.
**Description:**Genotypes used in this study.

**Title:** Supplementary Data 4.
**Description:** Oligos used in this study.
