# Supplementary material for: Transcriptional read-through of the long non-coding RNA SVALKA governs plant cold acclimation
Source: Nat Commun. 2018 Nov 1;9:4561. doi: 10.1038/s41467-018-07010-6 (PMC6212407; doi:10.1038/s41467-018-07010-6)
Supplement: Supplementary file 9 — Reporting Summary [file 41467_2018_7010_MOESM9_ESM.pdf]

nature research

Corresponding author(s): Sebastian Marquardt

Reporting Summary

Nature Research wishes to improve the reproducibility of the work that we publish. This form provides structure for consistency and transparency in reporting. For further information on Nature Research policies, see Authors & Referees and the Editorial Policy Checklist.

Statistical parameters

When statistical analyses are reported, confirm that the following items are present in the relevant location (e.g. figure legend, table legend, main text, or Methods section).

n/a	Confirmed	
☐	☒	The exact sample size (*n*) for each experimental group/condition, given as a discrete number and unit of measurement
☐	☒	An indication of whether measurements were taken from distinct samples or whether the same sample was measured repeatedly
☐	☒	The statistical test(s) used AND whether they are one- or two-sided<br>*Only common tests should be described solely by name; describe more complex techniques in the Methods section.*
☒	☐	A description of all covariates tested
☒	☐	A description of any assumptions or corrections, such as tests of normality and adjustment for multiple comparisons
☐	☒	A full description of the statistics including central tendency (e.g. means) or other basic estimates (e.g. regression coefficient) AND variation (e.g. standard deviation) or associated estimates of uncertainty (e.g. confidence intervals)
☒	☐	For null hypothesis testing, the test statistic (e.g. *F*, *t*, *r*) with confidence intervals, effect sizes, degrees of freedom and *P* value noted<br>*Give P values as exact values whenever suitable.*
☒	☐	For Bayesian analysis, information on the choice of priors and Markov chain Monte Carlo settings
☒	☐	For hierarchical and complex designs, identification of the appropriate level for tests and full reporting of outcomes
☒	☐	Estimates of effect sizes (e.g. Cohen's *d*, Pearson's *r*), indicating how they were calculated
☐	☒	Clearly defined error bars<br>*State explicitly what error bars represent (e.g. SD, SE, CI)*

Our web collection on statistics for biologists may be useful.

Software and code

Policy information about availability of computer code

Data collection	No code or software was used to collect data.
Data analysis	All new code used in this study can be found at [https://github.com/Maxim-Ivanov/Kindgren_et_al_2018].<br>The analysis use CAGEfightR v1.0.0. The software can be found at [https://bioconductor.org/packages/release/bioc/html/CAGEfightR.html]

For manuscripts utilizing custom algorithms or software that are central to the research but not yet described in published literature, software must be made available to editors/reviewers upon request. We strongly encourage code deposition in a community repository (e.g. GitHub). See the Nature Research guidelines for submitting code & software for further information.

Data

Policy information about availability of data

All manuscripts must include a data availability statement. This statement should provide the following information, where applicable:

- Accession codes, unique identifiers, or web links for publicly available datasets
- A list of figures that have associated raw data
- A description of any restrictions on data availability

TSS-seq data is available at NCBI (GSE113677). TSS-seq analysis used computational methods. The scripts are available at github: [https://github.com/Maxim-

Ivanov/Kindgren_et_al_2018]. The authors declare that all other data supporting the findings of this study are available from the corresponding author upon request. The source data underlying all figures can be found in the Sourde Data file.

Field-specific reporting

Please select the best fit for your research. If you are not sure, read the appropriate sections before making your selection.

☒ Life sciences ☐ Behavioural & social sciences ☐ Ecological, evolutionary & environmental sciences

For a reference copy of the document with all sections, see nature.com/authors/policies/ReportingSummary-flat.pdf

Life sciences study design

All studies must disclose on these points even when the disclosure is negative.

Sample size	All experiments were done with at least three biological replicates except the TSS-seq, which was done with 2 biological replicates.
Data exclusions	No data was excluded
Replication	Biological replicates of plant samples were grown on separate plates and treated as independent samples throughout all experimental procedures.
Randomization	No randomization was used in this study
Blinding	No blinding was used in this study

Reporting for specific materials, systems and methods

Materials & experimental systems

n/a	Involved in the study	
☒	☐	Unique biological materials
☐	☒	Antibodies
☒	☐	Eukaryotic cell lines
☒	☐	Palaeontology
☒	☐	Animals and other organisms
☒	☐	Human research participants

Methods

n/a	Involved in the study	
☒	☐	ChIP-seq
☒	☐	Flow cytometry
☒	☐	MRI-based neuroimaging

Antibodies

Antibodies used	Anti-FLAG (Sigma-Aldrich, Cat no: F3165), Anti-NRPB1 (abcam, Cat no: ab140509), Anti-RNAPII 8WG16 (abcam, Cat no: ab817)
Validation	Anti-FLAG: Validated for Arabidopsis. Validated for Western and immunoprecipitation<br>Anti-NRPB1: Predicted to react with plant NRPB1. Validated for Western.<br>Anti-RNAPII 8WG16: Validated for Arabidopsis. Validated for Western.
